# Supplementary material for: End Sequence Analysis Toolkit (ESAT) expands the extractable information from single-cell RNA-seq data
Source: Genome Res. 2016 Oct;26(10):1397–410. doi: 10.1101/gr.207902.116 (PMC5052061; doi:10.1101/gr.207902.116)
Supplement: Supplemental Material [file supp_gr.207902.116_Supp_Materials.tar.gz › ESAT-esat_v0.1/doc/html/classbroad_1_1pda_1_1gene_1_1_gene_tools-members.html]

ESAT: Member List


ESAT


- Main Page
- Related Pages
- Packages
- Classes
- Files

- Class List
- Class Index
- Class Hierarchy
- Class Members

All Classes Namespaces Files Functions Variables Enumerator Pages

- broad
- pda
- gene
- GeneTools

broad.pda.gene.GeneTools Member List

This is the complete list of members for broad.pda.gene.GeneTools, including all inherited members.

|  |  |  |
| --- | --- | --- |
| intersectGeneSets(Collection< Gene > set1, Collection< Gene > set2, boolean takeComplement) | broad.pda.gene.GeneTools | static |
| main(String[] args) | broad.pda.gene.GeneTools | static |
| merge(Map< String, Collection< Gene >> originalMap) | broad.pda.gene.GeneTools | static |
| merge(Collection< Gene > original) | broad.pda.gene.GeneTools | static |
| mergeByGtfGeneId(String inFile, String outFile, String outFormat, String src, boolean writeMode) | broad.pda.gene.GeneTools | static |
| mergeSets(Map< String, IntervalTree< Gene >> set1, Map< String, IntervalTree< Gene >> set2) | broad.pda.gene.GeneTools | static |
| orientationForGene(Gene g, Sequence cSeq) | broad.pda.gene.GeneTools | static |
| orientationFromSpliceSites(Annotation introns, Sequence seq, boolean onlyCanonical) | broad.pda.gene.GeneTools | static |
| orientationFromSpliceSites(Annotation introns, Sequence chrSeq) | broad.pda.gene.GeneTools | static |
| overlap(Map< String, IntervalTree< Gene >> set1, Map< String, IntervalTree< Gene >>set2, Boolean byExon) | broad.pda.gene.GeneTools | static |
| selectHighScoringIsoform(Gene transcript, BEDFileParser set) | broad.pda.gene.GeneTools | static |


---

Generated on Fri Aug 15 2014 15:10:59 for ESAT by  

 1.8.7
